# N-Pep-Zn Improves Cognitive Functions and Acute Stress Response Affected by Chronic Social Isolation in Aged Spontaneously Hypertensive Rats (SHRs)

**DOI:** 10.3390/biomedicines12102261

**Published:** 2024-10-04

**Authors:** Mikhail Y. Stepanichev, Mikhail V. Onufriev, Yulia V. Moiseeva, Olga A. Nedogreeva, Margarita R. Novikova, Pavel A. Kostryukov, Natalia A. Lazareva, Anna O. Manolova, Diana I. Mamedova, Victoria O. Ovchinnikova, Birgit Kastberger, Stefan Winter, Natalia V. Gulyaeva

**Affiliations:** 1 Laboratory of Functional Biochemistry of the Nervous System, Institute of Higher Nervous Activity and Neurophysiology, Russian Academy of Sciences, 117485 Moscow, Russia; 2 Research and Clinical Center for Neuropsychiatry of Moscow Healthcare Department, 115419 Moscow, Russia; 3 Ever Pharma, Oberburgau 3, 4866 Unterach am Attersee, Austria

**Keywords:** N-Pep-Zn, SHRs, aging, chronic social isolation, learning, memory, stress response, cognitive, sympathetic, hypothalamo–pituitary–adrenal axis

## Abstract

Background/Objectives: Aging and chronic stress are regarded as the most important risk factors of cognitive decline. Aged spontaneously hypertensive rats (SHRs) represent a suitable model of age-related vascular brain diseases. The aim of this study was to explore the effects of chronic isolation stress in aging SHRs on their cognitive functions and response to acute stress, as well as the influence of the chronic oral intake of N-Pep-Zn, the Zn derivative of N-PEP-12. Methods: Nine-month-old SHRs were subjected to social isolation for 3 months (SHRiso group), and one group received N-pep-Zn orally (SHRisoP, 1.5 mg/100 g BW). SHRs housed in groups served as the control (SHRsoc). The behavioral study included the following tests: sucrose preference, open field, elevated plus maze, three-chamber sociability and social novelty and spatial learning and memory in a Barnes maze. Levels of corticosterone, glucose and proinflammatory cytokines in blood plasma as well as salivary amylase activity were measured. Restraint (60 min) was used to test acute stress response. Results: Isolation negatively affected the SHRs learning and memory in the Barnes maze, while the treatment of isolated rats with N-Pep-Zn improved their long-term memory and working memory impairments, making the SHRisoP comparable to the SHRsoc group. Acute stress induced a decrease in the relative thymus weight in the SHRiso group (but not SHRsoc), whereas treatment with N-Pep-Zn prevented thymus involution. N-pep-Zn mitigated the increment in blood cortisol and glucose levels induced by acute stress. Conclusions: N-pep-Zn enhanced the adaptive capabilities towards chronic (isolation) and acute (immobilization) stress in aged SHRs and prevented cognitive disturbances induced by chronic isolation, probably affecting the hypothalamo–pituitary–adrenal, sympathetic, and immune systems.

## 1. Introduction

The rapid aging of the world’s population presents one of the most significant challenges that we face today. Aging is a universal phenomenon, characterized by a multifaceted biological process that induces various changes across all organs and tissues of living beings. The brain, in particular, is among the organs most affected by aging. In both humans and animal models, age-related cognitive decline is associated with changes in specific brain structures. The aging brain exhibits alterations at multiple levels, from molecular and cellular levels to the level of networks, all of them contributing to adverse modifications in neuroplasticity, and thus negatively influencing learning and memory. Aging promotes cognitive decline and is a major risk factor for the development of common neurological diseases [1,2]. As the life expectancy continues to increase worldwide, age-related cognitive disturbances are becoming increasingly impactful on society and are likely to pose even greater challenges in the future. Numerous studies have reported significant cognitive variability among aged individuals, with chronic stress exposure throughout life being one of the factors influencing cognitive preservation. Chronic exposure to stress hormones impacts brain structures involved in cognition and other aspects of mental health, which may be particularly important in the aging brain due to its increased vulnerability to extreme factors [3,4].

In the 1990s, several studies established spontaneously hypertensive rats (SHRs) as a model for aging-related comorbid pathologies based on age-dependent hypertension [5,6]. Although normotensive at birth, SHRs develop sustained hypertension by 6 months of age. SHRs exhibit compensated cardiac hypertrophy, with eventual transition to heart failure in the final quartile of their lifespan; this makes them a valuable model for studying the progression from stable compensated hypertrophy to decompensated heart failure in the context of aging [7]. Aged SHRs naturally develop a reduced coronary flow reserve, an increased coronary vascular resistance, cardiac fibrosis, and impaired cardiac function, along with glomerular hypertension and ischemia, proteinuria, glomerular sclerosis, and interstitial fibrosis, similar to what is observed in hypertension in humans [8]. Hypertension is a critical risk factor for cerebrovascular diseases, including stroke, and is involved in the development of vascular cognitive impairment (VCI) and vascular dementia (VD). SHRs are widely regarded as the gold standard animal model for studying the association between hypertension and VCI/VD. Cerebrovascular changes, brain atrophy, the loss of nerve cells in cerebrocortical areas, and glial reactions have been documented in SHRs [9], with several changes resembling those found in in vivo imaging studies of patients with essential hypertension. Microanatomical, neurochemical, and behavioral data on SHRs suggest that this strain is a suitable model for age-related vascular brain diseases [10,11].

Age-associated cognitive disturbances, including dementia and mild cognitive impairment, necessitate effective treatments, making the development of drugs that improve and preserve cognition in the elderly a critical priority [12]. Various molecules with neuroprotective properties have been proposed, including neuropeptides. A number of peptide drugs with potential cognitive benefits are currently available, most of which are administered parenterally due to poor absorption from the gastrointestinal tract. Since peptide drugs are typically prescribed for chronic conditions, the need for continuous, repetitive daily injections presents a significant disadvantage compared to the oral route, which is associated with higher patient compliance [13].

N-Pep-Zn is a formulation based on N-PEP-12, a compound derived from purified neuronal proteins. N-PEP-12 contains peptides that have been shown to support brain function, particularly in the context of aging. These peptides promote neuronal survival, stimulate neurite outgrowth, and protect against metabolic stress, as evidenced in various experimental models [14]. In N-PEP-Zn, zinc is added to N-PEP-12 to enhance its potential cognitive benefits. Zinc, an essential trace element, plays a crucial role in synaptic plasticity and neurotransmission, further contributing to the preservation of cognitive function in aging populations [15]. This formulation combines the neuroprotective effects of N-PEP-12 with the well-known cognitive support provided by zinc [16].

The aim of this study was to explore the effects of chronic isolation stress in aging SHRs on their cognitive functions and stress response, as well as the impact of the chronic oral administration of N-Pep-Zn, the zinc derivative of N-PEP-12 [16]. This study is the first experimental investigation with N-PEP-Zn in vivo.

## 2. Materials and Methods

### 2.1. Animals

**Figure 1:**
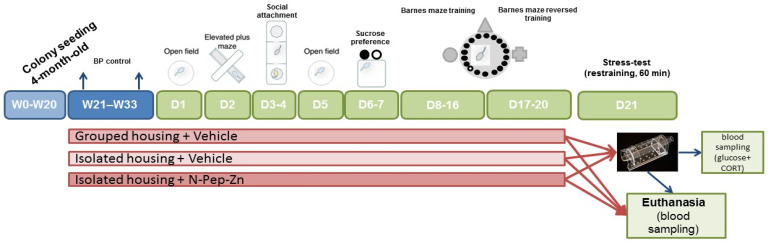
Experimental protocol.

Fifty-two male SHRs were supplied by “Pushchino” animal farm (Branch of the M.M. Shemyakin and Yu.A. Ovchinnikov Institute of Bioorganic Chemistry of the Russian Academy of Sciences (BIBCh RAS), Moscow, Russia) at the age of 4 months. BIBCh RAS purchased SHRs from Charles River Laboratories (Wilmington, MA, USA); the rats were housed under standard conditions at The Animal Breeding Facility (the Unique Research Unit Bio-Model of the IBCh RAS) before being brought to the Institute of Higher Nervous Acivity and Neurophysiology RAS (IHNA RAS). From the age of 4 months, the animals were housed in the institutional colony room of IHNA RAS with 5 per a cage under the conditions of 12 h light/dark cycle (light on 8:00). Food and water were available ad libitum. The experimental design is presented in Figure 1 and described below.

### 2.2. Social Isolation Housing

Nine-month-old animals were divided into two groups. Two thirds of the animals were housed in individual nontransparent cages (120 × 120 × 200 mm) while the remaining stayed in their home cages (480 × 375 × 210 mm) in groups of 2–3 rats. Food and water were available ad libitum. Isolated and socialized rats were housed in the same room but near the opposite walls. The animals were isolated for 3 months. Within this period, the animals remained undisturbed except for a short period of day time when they received treatment, sawdust was replaced, and blood and saliva sampling procedures were carried out as indicated in Figure 1. Body weight was controlled weekly as a measure of the physiological state of the animals.

### 2.3. N-Pep-Zn Preparation and Treatment

N-Pep-Zn powder (Ever Pharma, Unterach, Austria) was dissolved in water (1316 mg in 34 mL) with stirring. The pH of the solution was adjusted to 7.0–7.2 by addition of 1 M NaOH. The solution was centrifuged at 4000 rpm for 10 min and the supernatant was filtered. To ensure microbiological stability and sterility we filtered the solutions using a 0.22 µm syringe membrane filter. This step was done as the prepared solutions were aliquoted and stored frozen for later use throughout the course of the experiment, and filtration through a 0.22 µm membrane filter was applied as a standard procedure to remove any potential microbial contaminants, thus preventing bacterial growth and ensuring that the solution remained sterile and safe for use over time. Then, the solution was aliquoted and kept frozen at −18 °C. The animals were treated with N-Pep-Zn solution at a dose of 40 µL per 100 g of body weight daily for 3 months.

The vehicle consisted of 46 mg NaCl and 550 mg lactose dissolved in 35 mL H_2_O. After filtration, the solution was aliquoted and kept frozen at −18 °C. The animals were treated with this solution at a dose of 40 µL per 100 g of body weight.

For the treatment, isolated animals were divided into two subgroups, one of each was treated with the N-Pep-Zn solution whereas the other subgroup was treated with the control solution. The socialized rats were treated with the control solution. Both solutions were given per os daily during the whole period of isolation.

### 2.4. Blood Pressure Measurement

Before the start of the isolation period and after 3 months of isolation, blood pressure was measured using the tail cuff method. For this purpose, the animals were adapted to plastic holders, which were used for the procedure, for 3 days. During the measurement, the rat was placed into the holder and a cuff was put onto the tail. The holder was placed onto a “Flogiston” heating plate (Neurobotics, Moscow, Russia) to maintain a stable body temperature. The cuff was connected to a computer-assisted “Sistola” device (Neurobotics, Moscow, Russia). Blood pressure was measured using original software supplied by a manufacturer. Three measurements were conducted with a 15 min interval between them. The mean value was calculated and used as an index of the blood pressure in the animal.

### 2.5. Behavioral Study

For behavioral tests we used apparatuses supplied by “Open Science Ltd.” (Krasnogorsk, Russia). Animal behavior was recorded with DMK 23GV024 GigE camera connected to a personal computer, using IC-Capture Ver. 2.2.248.1000 software (The Imaging Source Europe GmbH, Bremen, Germany). Behavioral tracing and analyses were performed using EthoVision 11XT software (Noldus, Wageningen, The Netherlands). Thirty minutes before each behavioral test (except for the sucrose preference test), the animals were transported into the behavioral room in their home cages and adapted to behavioral conditions.

#### 2.5.1. Sucrose Preference Test

The sucrose preference test (SPT) is often used to detect depressive-like features in animals [17]. For this purpose, the rats from the social control group were placed individually into clear Plexiglas cages to decrease the effect of individual housing. The isolated animals were tested in cages made of nontransparent plastic. The cages were located in a room separated from the colony room. The test was performed as previously described [18]. On day 1, each rat was exposed to two drinking bottles filled with fresh water. On the next day, water in one of the bottles was replaced with 2% sucrose solution. Then, drinking behavior was observed for 48 h. The positions of the bottles were changed every 12 h. Food pellets were available for the whole period of observation. Bottles with water or sucrose solution were weighed before the start and after the end of each 12 h interval. Sucrose consumption was calculated as total weight of consumed solution. Sucrose preference was calculated as sucrose weight, which referred to total weight of consumed liquid (sucrose solution + water), and presented as a percentage.

#### 2.5.2. Open Field Test

The “Open field test” (OFT) was used to estimate locomotor and exploratory activity, anxiety, and emotionality in rats [19,20]. The OFT was performed in a gray circular arena with a diameter of 100 cm surrounded by a 30 cm high wall. The arena was situated in the center of a sound-protected room. The floor of the arena was evenly lit (450 lx) with four diode lamps located on the ceiling of the room. The rat was placed into the center of the arena and its behavior was recorded for 5 min.

#### 2.5.3. Elevated Plus Maze

The “Elevated plus maze” (EPM) test was developed to test the anxiolytic effects of drugs and is widely used for pharmacological experiments and analysis of anxiety in experimental animals under conditions of variable stress; it incorporates a free choice of safety conditions and allows one to assess the level of anxiety of the animal by preference of dark/light area, fear of height, and the severity and time course of the “looking out” behavior [21,22]. The EPM consisted of four crossed arms with a size of 50 × 14 cm. Two closed arms had side walls that were 30 cm high, and the open arms had side boards of 1 cm height. The intensity of open arms illumination was 300 lx. The rat was placed onto the central platform facing an open arm. For 5 min the latency to start movement, number of entries into and time spent in the open arms and closed arms, number of head deeps and stretching postures, and grooming and defecation boli were recorded.

#### 2.5.4. Three-Chamber Sociability and Social Novelty Test

The three-chamber sociability and social novelty test allows researchers to estimate cognition in the form of sociability and interest in social novelty in rodents. A chamber (120 × 80 × 40 cm) was divided onto three equal compartments by two clear partitions. In the center of each partition there was an opening (10 × 10 cm) closed by a guillotine door. The central compartment was empty whereas, in both side compartments, cylinder baskets (20 cm in diameter and 30 cm high) were located. The 10 min habituation session started by placing the rat into the central compartment; the doors were opened immediately after the placement. After the end of the habituation the rat was returned into the central compartment. The never-before-met Stranger 1 rat was introduced into one of the baskets and the 10 min sociability session was initiated by opening the doors. In this session, the rat was allowed to explore both the empty compartments and the compartment with a new subject. After the session had elapsed, the rat was returned into the central compartment and the doors were closed. The never-before-met Stranger 2 rat was introduced into the other basket and the baskets were alternated between the side compartments. Then, the doors were opened and the rat was allowed to explore both side compartments with Stranger 1 and the Stranger 2 during the next 10 min social novelty session. The behavior of rats was video recorded and then the time spent sniffing each basket, the time spent in each compartment, and the number of entries into each compartment were evaluated.

#### 2.5.5. Spatial Learning and Memory in a Barnes Maze

In order to assess spatial learning and memory in animals, we trained them to find a hide shelter in a Barnes maze. The protocol suggested by [23] was applied. The maze was a circular arena with a diameter of 122 cm. Eighteen holes with a diameter of 9.5 cm were located along the edge of the maze at an equal distance from each other and from the center of the maze. Seventeen holes were closed with false shelters which did not allow the rats to escape the maze. One of the holes was connected to a true shelter, such as a box (11 × 30 cm). The maze was mounted 113 cm above the floor. The arena was evenly brightly lit (500 lx). A speaker was located 130 cm over the center of the maze. External cues, such as contrast black and white geometric figures (60 × 60 cm), were located around the maze at a distance of 60 cm from and 60 cm above the arena level. During training, the shelter was located at the same place in the maze for all animals. The training was preceded by an adaptation trial, where the rat was released from a box in the center of the arena and allowed to explore the maze for 5 min. Then, the rat was trained to find the hidden shelter for 5 days with two trials per day and 20 min intervals between the trials. Before the trial, the rat was placed into the maze under the unclear box. Twenty seconds later, the box was raised and the rat was allowed to attempt to find the entry into the hidden shelter for 3 min. If the rat did not find the shelter during this time, it was directed towards the shelter with light guiding movements. To speed up the animal’s departure from the center of the maze, a white noise of 90 dB was used, which was emitted by a speaker located above the arena. After the animal entered the shelter, the noise stopped. The rat was left in the shelter for 1 min and then returned to the home cage. The second trial was conducted in a similar manner. The surface of the maze and the shelter were cleaned with water and 70% ethanol between the trials and the maze was turned in a random direction to face a random angle in order to prevent the finding of the shelter using olfactory marks, while the shelter remained in the same position. Twenty-four hours (h) after the last training trial, test trial 1 was performed. In the test trial, the shelter was replaced by a false shelter, the rat was placed in the center of the arena as described above, and the rat’s behavior was video recorded for 60 s. During the training, the latency to find the shelter was used as an index of learning. During the testing, the time spent in the target sector was used as an index of long-term memory, and the time spent in the opposite sector was used to assess the capability of the rat to differentiate between the target and non-target locations in the maze.

Twenty-four hours after test trial 1, the reversed training in the Barnes maze was performed. It consisted of a three-day session with two trials per day. During the reversed training, the shelter was relocated into the position opposite to that in the initial training session. The training procedure was similar to that described above. After the end of this training session, test trial 2 was conducted, in which the hidden shelter was replaced with the false shelter. The same learning and memory indices were recorded.

### 2.6. Collection of Biomaterial

#### 2.6.1. Blood Collection

Blood from the caudal vein was collected under brief isoflurane anesthesia before, in the middle of, and after the end of the 3-month isolation period, no later than 3 days before the start of the behavioral session. Blood was also collected at the time point 36 weeks before, as well as under, acute immobilization stress (100 µL; 0, 30 min, 60 min, the animals were immobilized). After acute stress, the anaesthetized animals were instantly decapitated and, post-decapitation, blood was also collected. Blood was centrifuged for 15 min at 1500 g at 4 °C to obtain plasma, which was aliquoted and stored at −80 °C before biochemical studies.

#### 2.6.2. Saliva Collection

Rat saliva samples were collected according to the method described by Guhad and Hau [24] with minor modifications. Small squares of laboratory filter paper (5 × 5 mm) were put into the mouths of the anaesthetized rats. After 5 min, the squares were removed, immersed in 100 μL sterile 0.9% NaCl in Eppendorf tubes, and stored at −80 °C. Saliva samples were collected before isolation (20 weeks), during isolation (27 weeks and 33 weeks) and after acute immobilization stress (36 weeks; all terms correspond to respective time points in Figure 1).

### 2.7. Measurements of Biochemical Indices

#### 2.7.1. Enzyme-Linked Immunosorbent Assays (ELISA)

To determine the plasma corticosterone levels, kits for the enzyme-linked immunosorbent assay (Kit Corticosterone for 96 tests, cat. № K210R, Xema, Moscow, Russia) were used; the kits allow us to detect both free and transport protein-bound corticosterone by a competitive ELISA method.

The levels of the proinflammatory cytokines IL1β and IL6, and TNFα in blood plasma of rats were measured by R&D Systems Quantikine ELISA Kits (R&D Systems, Inc., Minneapolis, MN, USA) according to the manufacturer’s instructions (cat. № DY501; cat. № SR6000B; cat. № SRTA00).

The levels of C-reactive protein and TGFβ in blood plasma of rats were measured by R&D Systems Quantikine ELISA Kits according to the manufacturer’s instructions (cat. № DY1744; cat. № DB100C).

#### 2.7.2. Salivary Protein Concentration and Salivary Amylase Activity

The protein concentration in the saliva was determined by Bradford method (Coomassie (Bradford) Protein Assay kit, cat. № 103162, Thermo Fisher Scientific, Waltham, MA, USA) and salivary amylase activity was measured by the kinetic method at 405 nm, using 2-chloro-p-nitrophenyl-α-D-maltotrioside as substrate (Kit α-Amylase, cat. № B-8059; Vector-Best-Europe, Moscow, Russia).

### 2.8. Statistical Analysis

The data were checked for normality using the Shapiro–Wilk W-test. Body weight changes were estimated using analysis of variances (ANOVA) with repeated measures (ANOVA-RM) using the experimental condition as the between-group and the week as the within-group factors, which was followed by post-hoc Tukey HSD tests. Most behavioral data did not correspond to normal distribution. Therefore, we applied Friedman ANOVA on ranks to analyze learning curves in the Barnes maze followed by Wilcoxon matched-pair test for within-group comparisons and Mann–Whitney U test for between-group comparisons. Wilcoxon matched-pair test was used to analyze latencies in long-term memory tests. Non-parametrical methods were used to analyze animal behavior in the other tests. For statistical analysis of data on CORT and glucose, ANOVA-RM was used followed by post-hoc Tukey HSD tests. Specific statistical tests are indicated in the text or figure captions. The results are presented as median ± interquartile range or M ± s.e.m. as indicated in figure captions. The level of significance *p* = 0.05 was accepted.

## 3. Results

### 3.1. Physiological Indices

#### 3.1.1. Mortality

Mortality from unknown reasons occurred for 2 of 19 rats in the SHRsoc group (10.5%), 5 of 16 rats in the SHRiso group (31.3%), and 2 of 17 rats in the SHRisoP group (11.8%). The highest mortality was observed in the SHRiso group, especially during the last weeks when the rats were subjected to behavioral testing and the rats reached the age of 12–13 months. Although apparently different, this difference was not significant, according to the two-tailed Fisher exact test (*p* = 0.21), as compared to the SHRsoc group. The differences between the SHRisoP and SHRsoc (*p* = 1.0) or SHRiso groups (*p* = 0.22) were not significant either. The final number of survived animals in the experimental groups was: *n* = 17 SHRsoc; *n* = 11 SHRiso; and *n* = 15 SHRisoP.

#### 3.1.2. General Condition

**Figure 2:**
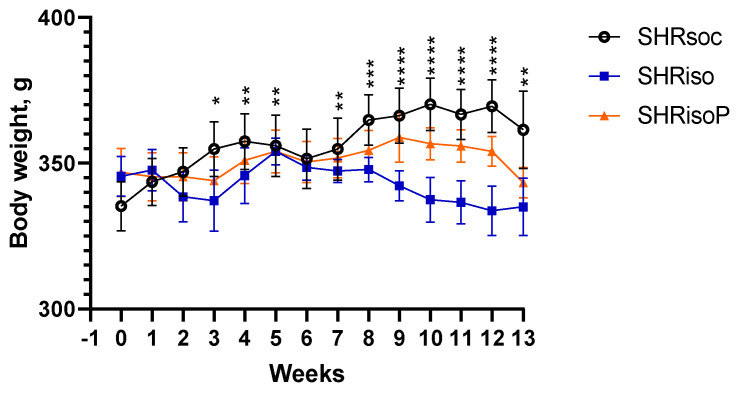
Body weight gain in the rats subjected to group or social isolation rearing conditions. Initial weighing (week 0) was performed before the start of social isolation period, when the rats were 9-months-old. Data are presented as M ± s.e.m. ANOVA results are presented in the text. The differences in the body weight were statistically significant as compared to week 0 at *—*p* < 0.05, **—*p* < 0.01, ***—*p* < 0.001, and ****—*p* < 0.0001) in the SHRsoc group only according to the Tukey HST test for multiple comparison of means. Here and in Figures 3–9, *n* = 17 SHRsoc; *n* = 11 SHRiso; *n* = 15 SHRiso.

The general conditions of the animals were estimated using their weekly weighing during the isolation period. Changes in the animals’ body weight are presented in Figure 2. The ANOVA for repeated measures revealed that there was no direct “treatment” effect (*F_2,49_* = 1.17, *p* = 0.32), whereas the effect of “time(week)” (*F_12,142_* = *p* < 0.05) and “treatment” × “time” interaction (*F_26,615_* = 3.43, *p* < 0.0001) were significant. The body weight increased in the SHRsoc group, whereas isolation prevented the body weight gain in both SHR groups subjected to chronic isolation stress (SHRiso, SHRisoP) (Figure 2).

#### 3.1.3. Arterial Pressure and Heart Rate

The arterial pressure was measured before the start and after the 3-month period of social isolation by the plethysmographic method. The blood pressure values in the three groups of rats, SHRsoc, SHRiso, and SHRisoP, did not differ from each other (205 ± 7, 203 ± 6, and 207 ± 5 mmHg, respectively), similarly to the heart rate (430 ± 5, 420 ± 8, and 442 ± 6 b.p.m, respectively), before the start of isolation. After the 3-month isolation, the arterial pressure values decreased in the SHRsoc, SHRiso, and SHRisoP groups (178 ± 6, 164 ± 8, and 163 ± 11 mmHg, respectively). The ANOVA-RMANOVA-RM revealed an effect of “week” (*F_1,44_* = 55.44, *p* < 0.0001), but no effect of “treatment” (*F_2,49_* = 0.39, *p* = 0.68) or “treatment” × “week” interaction (*F_2,44_* = 1.11, *p* = 0.34). However, the heart rate significantly increased after the isolation period up to 513 ± 14, 530 ± 17, and 508 ± 16 b.p.m. in the SHRsoc, SHRiso, and SHRisoP groups, respectively. The ANOVA-RM revealed a “treatment” × “week” interaction (*F_2,44_* = 44.13, *p* < 0.0001) and no effect of “week” (*F_1,44_* = 2.08, *p* = 0.15) or “treatment” (*F_2,49_* = 0.043, *p* = 0.96). We can assume that these changes were probably due to some general factors which influenced all the groups of rats studied, such as, probably, aging during the experiment (arterial pressure decrease) or repeated placement into restrainers used for pressure measurement (heart rate) after the long-term interval.

#### 3.1.4. Behavior

##### Locomotor and Exploratory Activity

The behavior in the OFT reflects general locomotion, movement, and exploration in laboratory rodents. We did not find significant effects of social isolation or N-Pep-Zn in the isolated rats on the distance traveled in the OFT and number of rearing events (Table S1). The rats of all groups exhibited similar activity, measured as the number of total arm entries in the EPM (Table S1).

##### Emotional State, Anxiety and Depression-like Behavior

The emotionality was estimated by the number of boli in the OFT, grooming, and the latency to start movement after the placement into the arena. The animals of all groups showed similar values for these indices. They did not differ in the number of boli, amount of stretching, number of grooming episodes, and risk assessment in the EPM either (Table S1).

Anxiety was assessed using the EPM. The rats of the SHRsoc, SHRiso, and SHRisoP groups did not differ in the time spent in the open or closed arms or the number of entries into those arms in the EPM. The ratio of the distance traveled in the center and periphery of the arena in the OFT, often used as an index of anxiety in rats, were similar in all groups (Table S1).

The SPT was used to estimate anhedonia in animals, i.e., an index of depression-like behavior. The rats of all groups consumed a similar volume of liquid, both water or sucrose solution, during the 48 h test. They also had a similar rate of sucrose preference (Table S1).

##### Social Behavior

The three-chamber test was applied to estimate the sociability and preference of social novelty in the SHRsoc, SHRiso, and SHRisoP groups of animals. Although the rats clearly demonstrated a trend toward sociability, preferring to spend more time in the compartment with a stranger rat as compared to in the empty compartment, no effect of social isolation or isolation + N-Pep-Zn was observed when comparing the SHRsoc, SHRiso, and SHRisoP groups. We did not find any effect of the experimental conditions on the social novelty indices either (Table S1).

Thus, we did not reveal any significant influence of social isolation on the indices of activity, exploration, anxiety, depression-like behavior, or social behavior in the aging SHRs. This may be the main reason why we did not observe any effects of N-Pep-Zn in these behavioral tests.

##### Learning and Memory

**Figure 3:**
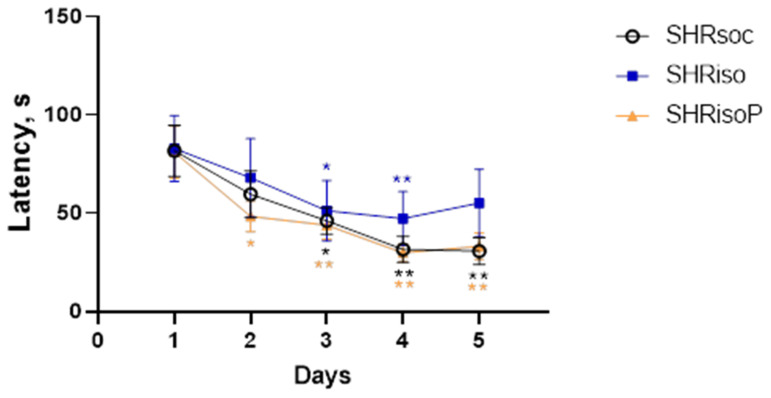
Changes in the latency to find a hidden shelter in the Barnes maze. Data are presented as M ± s.e.m. The differences are significant at *—*p* < 0.05 and **—*p* < 0.01 vs. the latency on day 1 according to the Wilcoxon test. Color of asterisks indicates the difference in the respective group.

Cognitive processes in rats were studied using the standard task in the Barnes maze. The latency to find a hidden shelter did not differ between the groups during the first session (Figure 3). The latency decreased in all three groups of animals (Table 1), but this decrement was different depending on the type of experimental treatment. Specifically, the latency decreased from day 3 to day 5 in the SHRsoc and from day 2 to day 5 in the SHRisoP groups compared to day 1, whereas, in the SHRiso group, it was lower on days 3 and 4, but did not show an additional decrease on day 5 (Table 2).

**Figure 4:**
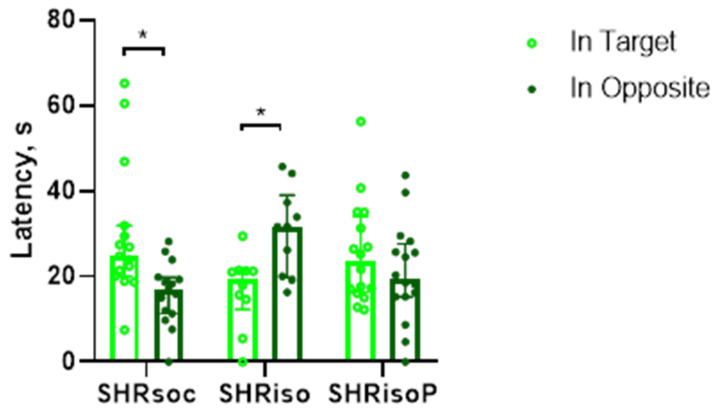
Effects of social isolation and N-Pep-Zn administration to isolated SHRs on long-term memory in the Barnes maze. The latency to stay in the target and opposite sectors of the maze during test trial 1 is presented. Data are presented as median values and first and third quartiles. The differences are significant at *—*p* < 0.05 according to Wilcoxon matched-pair test.

On day 6, test trial 1 was performed to study the long-term memory formed in the Barnes maze. During the trial, the animals had to recognize the position in which the hidden shelter was located during the training session. For this purpose, the surface of the maze was virtually divided into four sectors and the time spent in the target sector and the opposite sector was recorded. We did not find any significant differences between the groups in the time spent in the target sector of the maze during the test trials (Figure 4). However, only animals of the SHRsoc group preferred to spend more time in the target sector as compared to the opposite one. The rats subjected to social isolation did not preferentially recognize the target sector and even spent more time in the opposite sector. The treatment of isolated rats with N-Pep-Zn prevented this memory impairment, making the animals of the SHRisoP group more similar to the rats of the SHRsoc group.

**Figure 5:**
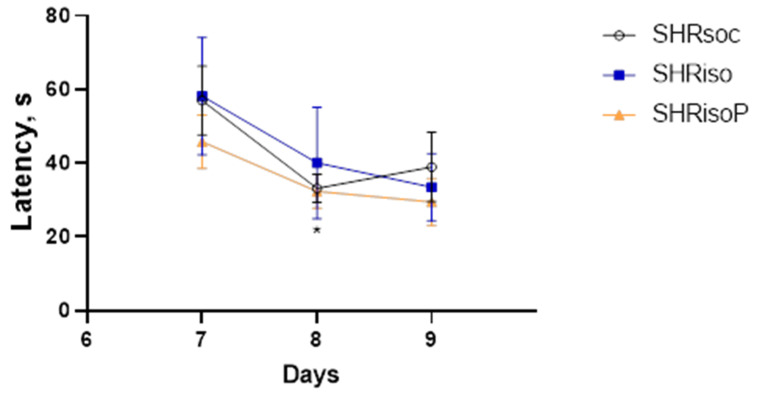
Changes in the latency to find a hidden shelter in the Barnes maze in the re-training session. Data are presented as M ± s.e.m. The difference is significant for the SHRsoc group vs. the latency on day 1 at *—*p* < 0.05 according to the Wilcoxon test.

To test cognitive flexibility, we re-trained the rats for an additional 3 days with the shelter located in the opposite sector to that during the first training session. During the re-training, the animals learned the new shelter location, but with lower efficiency (Figure 5). Despite the fact that the animals of all groups found the new location of the shelter during the first day of the re-training session, they exhibited a subtle latency decrement during the re-training. The ANOVA on ranks did not reveal any significant effect of training during days 7–9 (Table 3). The only difference was observed in the SHRsoc group on day 8 (the second day of re-training) as compared to day 7 (the first day of re-training); however, this decrease in the latency was not stable (Figure 5, Table 4).

**Figure 6:**
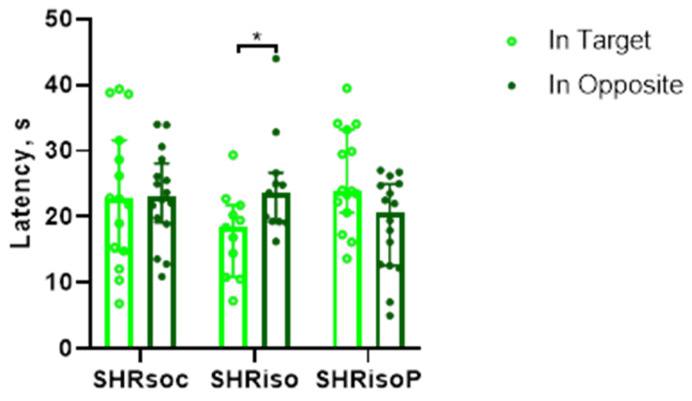
Effects of social isolation and N-Pep-Zn administration to isolated SHRs on long-term memory in the Barnes maze. The latency to stay in the target and opposite sectors of the maze during test trial 2 is presented. Data are presented as median values and first and third quartiles. The differences are significant at *—*p* < 0.05 according to Wilcoxon matched-pair test.

Similarly to test trial 1, there were no differences between the groups in the time spent in the target sector (Figure 6). However, the rats of the SHRiso group exhibited a reverted preference of the sector which was opposite to the target. This effect was not observed in either the SHRsoc or SHRisoP groups. However, the rats of the latter groups did not demonstrate any preference.

**Figure 7:**
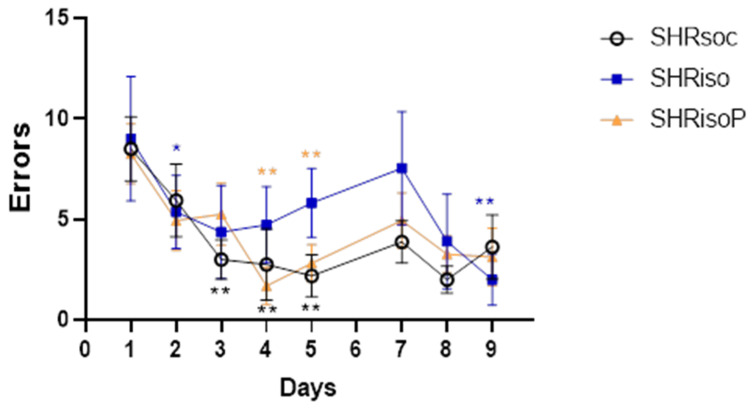
Effects of social isolation and N-Pep-Zn administration to isolated SHRs on working memory in the Barnes maze. The number of working memory errors during the training and reversal training is presented. Data are presented as median values and first and third quartiles. The differences are significant at *—*p* < 0.05 and **—*p* < 0.01 compared to day 1 of training or day 7 of reversal training according to Wilcoxon matched-pair test. Color of asterisks indicates the difference in the respective group.

Finally, we estimated the number of working memory errors made by rats during the training and re-training sessions. Repeated visits to the holes previously visited during the same trial were considered as working memory errors. The number of working memory errors progressively decreased in the SHRsoc group starting from day 3 of the training (Figure 7, Table 5).

In the SHRiso group, the number of working memory errors decreased only on day 2, but then the animals did not demonstrate any improvement of target-hole recognition, often visiting the holes previously visited in this trial. The treatment of isolated rats with N-Pep-Zn prevented the effects of isolated housing because these rats exhibited working memory capacities similar to those observed in the SHRsoc group.

During the reversal training, the most prominent decrease in the number of working memory errors was revealed in the SHRiso group, probably because these rats demonstrated a higher number of errors within both sessions as compared to the SHRsoc and SHRisoP groups.

Thus, N-Pep-Zn beneficially affected learning and memory in the Barnes maze. The chronically isolated SHRs did not prefer the maze quadrant with target holes, suggesting impairment of learning, while N-Pep-Zn mitigated this effect of social isolation. The reversal learning was ineffective in the SHRs, and chronic isolation induced preference of the opposite quadrant, suggesting the further impairment of learning/memory, while N-Pep-Zn induced preference of the target quadrant.

### 3.2. Biochemical INDICES

#### 3.2.1. Salivary Amylase Activity and Blood Inflammatory Markers

The alpha-amylase activity (an index of sympathetic activity) and total protein content in the saliva of the SHRs were measured before isolation and in the middle and at the end of the isolation period (W20, W27, and W33, respectively, Figure 1). The salivary alpha-amylase activity tended to increase during aging in the SHRsoc group; however, this effect was not statistically significant (3.00 ± 0.57; 4.26 ± 0.92; 6.86 ± 1.71 U/mg protein, respectively). This pattern was not observed in the SHRiso group (3.19 ± 0.97; 3.63 ± 0.94; 2.15 ± 0.48 U/mg protein, respectively), but recovered in the isolated SHR animals treated with N-Pep-Zn (3.30 ± 1.12; 3.98 ± 0.89; 6.05 ± 1.71 U/mg protein, respectively). The treatment of isolated rats with N-Pep-Zn showed a trend of stimulating protein secretion with saliva at the end of the isolation period (102.32 ± 9.52; 78.19 ± 8.31; 121.93 ± 15.15 μg/mL, respectively); this effect was not observed in the SHRsoc (92.41 ± 11.58; 71.21 ± 6.45; 83.37 ± 10.70 μg/mL, respectively) and SHRiso groups (91.15 ± 13.09; 92.23 ± 9.46; 89.93 ± 11.54 μg/mL, respectively).

After the end of the experiment, we assessed the levels of inflammatory markers in the blood of the rats. We did not reveal statistically significant differences in the levels of IL-1β, TNFα, TGF-β, and C-reactive protein in the groups of rats studied (Table S2). However, the experimental treatment significantly modified the level of IL-6 in the blood of the SHRs (H(3, 20) = 6.62, *p* < 0.05). Social isolation significantly decreased the IL-6 content in the blood of the SHRiso group (*p* < 0.05, Figure S1a), whereas the treatment with N-Pep-Zn partially recovered the IL-6 level until it coincided with the control level, although the IL-6 content in SHRisoP group did not significantly differ from that in SHRsoc and SHRiso groups.

#### 3.2.2. Effects of Social Isolation and N-Pep-Zn Treatment on the Response of SHRs to Acute Immobilization Stress

**Figure 8:**
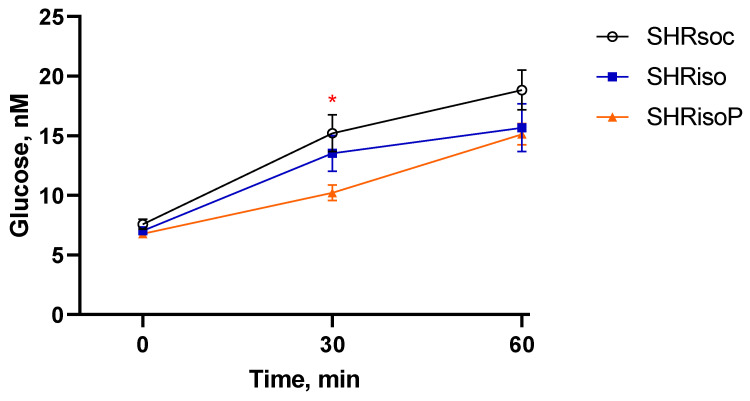
Changes in blood glucose level in the SHRsoc, SHRiso, and SHRisoP groups during the exposure to acute 1 h restraining. Data are presented as M ± s.e.m. The differences are significant in the SHRsoc between 0 and 30 min, 0 and 60, and 30 and 60 at *p* < 0.05, in the SHRiso between 0 and 30 and 0 and 60 min at *p* < 0.001, and in the SHRisoP group between 0 and 60 and 30 and 60 min at *p* < 0.01 according to Tukey HST test. *—*p* < 0.05 SHRsoc vs. SHRisoP, according to Tukey HST test.

After the end of the behavioral studies, the response of the rats to acute restraint stress was assessed. For this purpose, the rats were placed into restrainers for 1 h. The blood was sampled for analysis from the tail vein immediately after the placement (0 min), and after 30 and 60 min of stress exposure. We measured the glucose level in the peripheral blood as a general index of sympathetic activity and found that it increased in all the groups of rats studied (factor “Duration” *F_2,38_* = 109.90, *p* < 0.0001). However, the time course of the glucose increase had some specific features in different groups, as confirmed by the ANOVA with repeated measures (factor “Treatment” *F_2,18_* = 3.58, *p* < 0.05 and “Treatment” × “Duration” interaction *F_4,38_* = 2.83, *p* < 0.05). The changes in the blood glucose levels in the SHRsoc, SHRiso, and SHRisoP groups during the exposure to acute 1 h restraining are presented in Figure 8. N-Pep-Zn significantly smoothed the response of glucose to acute stress.

**Figure 9:**
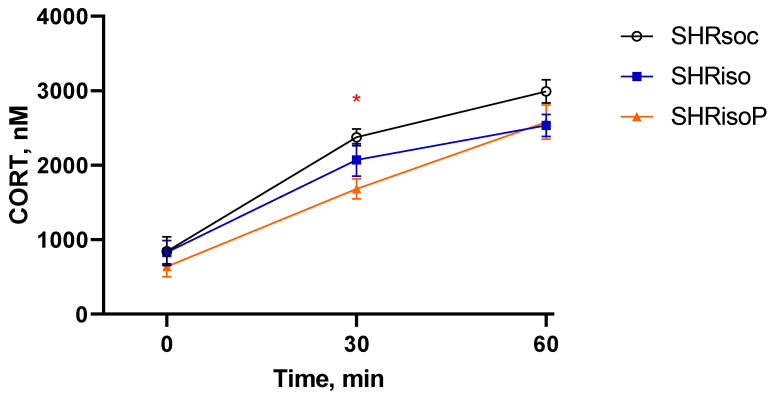
Changes in blood corticosterone (CORT) level in the SHRsoc, SHRiso, and SHRisoP groups during the exposure to acute 1 h restraining. Data are presented as M ± s.e.m. The differences are significant in the SHRsoc group between 0 and 30 min; 0 and 60 at *p* < 0.001, in the SHRiso between 0 and 30 and 0 and 60 min at *p* < 0.001, and in the SHRisoP group between 0 and 30, 0 and 60, and 30 and 60 min at *p* < 0.01 according to Tukey HST test. *—*p* < 0.05 SHRsoc vs. SHRisoP, according to Tukey HST test.

We also estimated the corticosterone level, a general index of the hypothalamo–pituitary–adrenal (HPA) axis, during the exposure to acute restraint stress. We found a corticosterone increase in all three groups of SHRs (factor “Duration” *F_2,38_* = 132.66, *p* < 0.0001; Figure 9). Similar to the glucose changes, the alterations in the corticosterone content were specific to the different groups (factor “Treatment” *F_2,18_* = 3.45, *p* = 0.053), although the interaction “Treatment” × “Duration” was insignificant (*F_4,38_* = 1.84, *p* = 0.14). The increment in the corticosterone level was lower in the SHRisoP group (Figure 9), similarly to that of glucose (Figure 8).

The glucose response was reduced in the SHRisoP group as compared to the SHRsoc group, probably indicating a lower sympathetic reactivity after the N-Pep-Zn treatment (Figure 8). Alterations in the salivary amylase activity, especially during stress, also reflect the sympathetic reactivity. We have measured the salivary amylase activity in the control and acutely stressed (immobilized) subgroups of the SHRsoc, SHRiso and SHRisoP groups but did not find significant effects of acute restraint stress on the amylase activity in the SHRsoc and SHRiso groups (Figure S1b). Interestingly, the amylase activity decreased in the stressed SHRisoP rats as compared to the control SHRisoP animals. Together with the observed lower glucose increments, these data may indicate reduced sympathetic reactivity to acute stress in animals subjected to chronic isolation.

After the end of the exposure to acute stress, we also estimated the levels of inflammatory markers in the blood of the SHRs. We did not reveal significant differences in the levels of IL-1β, TNFα, TGF-β, and C-reactive protein in the control or stressed subgroups of the SHRsoc, SHRiso, and SHRisoP animals studied (Table S2). Since the values of the IL-6 level and amylase activity did not correspond to normal distribution, non-parametrical statistical approaches were used (Figure S1). As mentioned above, chronic isolation significantly decreased the IL-6 level in the SHRs. The exposure of the isolated rats to acute restraint stress induced an increase in the IL-6 level in the SHRiso group (Figure S1a). The treatment of the isolated rats with N-Pep-Zn prevented the effect of chronic isolation on the basal level of IL-6, but also prevented an IL-6 response to acute restraint stress.

**Figure 10:**
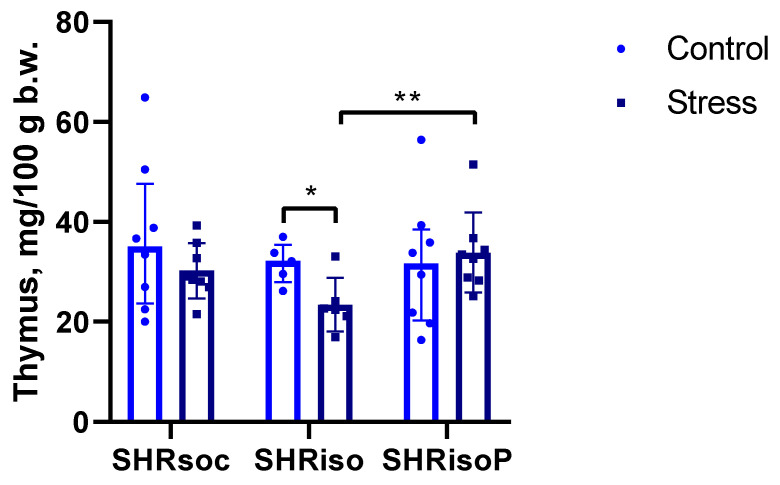
Effects of acute 1 h restraining on the relative thymus weight in the SHRsoc, SHRiso, and SHRisoP groups. Data are presented as median ± interquartile range. The differences are significant at *—*p* < 0.05 and **—*p* < 0.01 according to Mann–Whitney U test. *n* = 9 SHRsoc control; *n* = 8 SHRsoc restraint; *n* = 5 control; *n* = 6 SHRiso restraint; *n* = 7 SHRisoP control; *n* = 8 SHRisoP restraint.

Adrenal hypertrophy and thymus involution are among the essential components of the stress response. We compared the changes in the weight of the adrenals and thymus in the SHRsoc, SHRiso, and SHRisoP subgroups exposed to acute restraining stress to those in the animals of the respective control subgroups. We did not reveal considerable changes in the adrenals weights. However, significant differences were revealed in the thymus weights. The acute stress induced a decrease in the relative thymus weight (Figure 10), whereas treatment with N-Pep-Zn prevented this effect of stress in the isolated animals.

## 4. Discussion

SHRs are regarded as an animal model of genetic hypertension which, with aging, develops heart failure similarly to humans. The influence of the CNS on the cardiovascular system and blood pressure regulation was described in detail, including the role of the CNS in hypertension [25]. In studies of the myocardial tissue dysfunction mechanisms, a central role for neurohormonal activation, including the HPA axis and the renin–angiotensin–aldosterone system, was confirmed [26].

The hippocampus, a selectively vulnerable brain region, is sensitive to the effects of hypertension, and this has been confirmed in SHRs [11]. Overactivation of the HPA axis and the overproduction of glucocorticoid and mineralocorticoid neuroactive steroids in SHRs may be detrimental to the hippocampus, which is enriched in glucocorticoid and mineralocorticoid receptors. Dysfunction of the HPA axis in aging and age-associated diseases (diabetes, hypertension) induces hippocampal damage associated with impairments in learning, memory, and emotional sphere [27,28]. In aging and age-associated diseases, adrenocortical steroid overdrive sensitizes the hippocampus to the pathological milieu imposed by a pre-existing degeneration or illness [29]. Some abnormalities commonly found in the hippocampus of aging, diabetic, and hypertensive animals (in particular, SHRs) include decreased neurogenesis, astrogliosis and neuronal loss in the dentate gyrus, a low expression of brain-derived neurotrophic factor (BDNF), and a decreased number of neurons in the hilus [30]. In the hypothalamus, SHRs demonstrate increased expression of the hypertensinogenic peptide arginine vasopressin (AVP) and its V1b receptor. Hypertension and ageing upregulate the NO pathway in structures involved in the regulation of blood pressure in SHRs [31]. Alterations in neurotransmitter systems in SHRs have been reported, including abnormally low levels of dopamine in the neostriatum and nucleus accumbens [32]. Neurohumoral, neurochemical, and neurometabolic abnormalities in the brains of SHRs affect neuroplasticity, inducing learning and memory deficits and making this rat strain a clinically relevant model of dementia [11].

Vascular cognitive impairment dementia (VCID), an increasingly important cause of dementia in the elderly, embraces a number of diagnoses, including large vessel disease with multiple strokes, small vessel disease (SVD) with lacunar infarcts, and white matter disease. Because of its progressive course, SVD is thought to be the optimal form of VCID for treatment. Its pathophysiology involves hypoxic hypoperfusion resulting in injury to the white matter and neuronal death. Since hypertension leads to SVD, resulting in progressive damage to the white matter, neocortex, and hippocampus, SHRs may be regarded as a good model of SVD [33]. Importantly, similar ultrastructural breakdowns of cerebrocortical capillaries were revealed in Alzheimer’s disease, Parkinson’s disease, and SHRs, indicating that cerebral capillary damage is not exclusive to AD but occurs in other neurodegenerative disorders and hypertension as well. These ultrastructural abnormalities of cerebral capillaries may be causally related to decreased cerebral blood flow and create conditions which favor neurodegenerative mechanisms and eventually cause the development of dementia [34]. Thus, SHR is the rat strain most extensively investigated and used for assessing hypertensive brain damage and therapy. A high arterial blood pressure, brain atrophy, the loss of nerve cells, and glial reaction are phenomena shared with hypertensive brain damage in humans. Alterations the in neurotransmitter systems of SHRs are believed to have functional and behavioral relevance. In particular, the impaired cholinergic neurotransmission characteristic of SHRs is similar to that reported in patients affected by vascular diseases.

Communication is considered as one of the vital human requirements. Loneliness and social isolation are more than just a psychosocial problem. Chronic social isolation affects the clinical course of different diseases, including diabetes and cardiovascular and cerebrovascular diseases, as well as the average life expectancy and the risk of death caused by any causes, and is comparable with the effects of major risk factors like smoking, alcohol consumption, physical inactivity, hypertension, obesity, hypercholesterolemia, and various medical interventions [35]. The recent COVID-19-related social isolation and a more sedentary life in all age groups was most harmful for the elderly population who are the most vulnerable to infections and chronic neurodegenerative diseases. It is expected that the elderly patients with chronic neurodegenerative diseases who survived SARSCoV-2 infection will show aggravation of their neurodegenerative conditions [36,37]. Since social isolation and loneliness are believed to provoke consequent cognitive decline, specifically in the selectively vulnerable elderly population, we have chosen to use chronic isolation as a major challenge, a chronic stress inducing cognitive decline in SHRs, and explore the potential therapeutic effects of a new dietary peptide, N-Pep-Zn, on the behavioral and some biochemical indices of SHRs.

N-Pep-12, a compound consisting of biopeptides and amino acids, is the parent substance for N-Pep-Zn. N-Pep-12 is a dietary supplement with neuroprotective and pro-cognitive effects confirmed in experimental models and clinical studies [38]. In cultures, N-PEP-12 prevents the neuronal cell death of cortical neurons [39]. In animal experiments, the chronic administration of N-PEP-12 promotes neuronal plasticity in the limbic system of aged animals [40], partially reduces brain endothelial dysfunction [41], enhances cognitive function, and reduces neurodegenerative events associated with aging [42,43]. N-PEP-12 favors the neurorecovery of middle-aged and older adults with cognitive impairments after ischemic stroke [44,45,46] and may be an effective supplement to secure a healthy memory function in older adults [47]. The Zn-derivative of N-PEP-12 used in this study, N-Pep-Zn [16], is a novel development combining the beneficial effects of the N-PEP-12 peptides with the physiologically essential properties of zinc for proper CNS function [15].

Although chronic isolation increased the mortality of the SHRs during the experiment threefold (31.3% in SHRiso vs. 10.5% in SHRsoc), this difference, though impressive, was not statistically significant. The same is true for the beneficial effect N-Pep-Zn; although the mortality in the SHRisoP group (11.8%) was similar to that in the SHRsoc group, this striking effect could not be confirmed by statistical analysis, possibly due to the limited sample size of this study. The differences in mortality were more expressed in the aging of the 12–13-month-old SHRs. Importantly, in the surviving SHRiso animals, the effect of chronic isolation on the behavior of the SHRs was surprisingly diminutive. Indeed, we did not find a major influence of chronic social isolation on the indices of activity, exploration, anxiety, depression-like behavior, and social behavior in the aging SHRs (Table S1). This suggests that, in spite of the obviously pathological phenotype, the surviving SHRs were highly resilient to a chronic stress of this nature. Moreover, this may have been the expression of extensive multi-level adaptive mechanisms that allows this strain to survive in the situation of high arterial pressure and neurohumoral disturbances providing resistance to some other types of stress. We did not observe an effect of N-Pep-Zn in these behavioral tests, and the absence of effects of chronic isolation may be the main reason for this. It should be stressed that N-Pep-Zn did not exert adverse effects on physiological indices either.

The stability of major inflammatory markers in the SHRs subjected to chronic isolation (Table S2) confirms the resilience of SHRs to this kind of chronic stress. Interestingly, the SHR is an experimental model of salivary hypofunction, and these rats show a lesser flow and salivary protein concentration as well as lower dental mineralization [48,49]. Loneliness and chronic social stress may lead to activation of the sympathetic nervous system and reduced activity of the parasympathetic nervous system with enhanced sympathetic reactivity and/or delayed sympathetic recovery after exposure to an acute stressor [50]. The salivary alpha-amylase activity is a suitable measure of change in the sympathetic tone after the action of a psychosocial stressor, although it is not strongly related to the other indices of a stress response [51]. Chronic isolation abolished the normal pattern of salivary amylase in the SHRs, which tended to increase during aging and isolation in the SHRsoc group, but was restored in the isolated SHRs receiving N-Pep-Zn.

An important result of this study was the fact that social isolation did affect the cognitive function of the SHRs, and this was confirmed in the experiments with learning and memory in the Barnes maze (Figure 3, Figure 4, Figure 5, Figure 6 and Figure 7, Table 1, Table 2, Table 3, Table 4 and Table 5). Clear evidence for impairments in learning and memory was found in the SHRiso group as compared with the SHRsoc group. N-Pep-Zn prevented isolation-induced cognitive decline according to indices of learning and memory in the Barnes maze. Chronically isolated SHRs did not prefer the maze quadrant with target holes, while N-Pep-Zn diminished this effect of social isolation, bringing these animals close to the SHRsoc group. The reversal learning appeared unsuccessful in the SHRs, and this may reflect worse learning abilities, as reported by many groups. However, chronic isolation specifically provoked a preference for the opposite quadrant, confirming the further impairment of cognitive function in the SHRiso group, whereas N-Pep-Zn intake stimulated the preference for the target quadrant. Thus, in aged SHRs, the N-Pep-Zn significantly improved the spatial learning and memory affected by chronic social isolation.

Chronic isolation stress is able to modify the humoral and cellular immunity in rodents, although the data on this are quite contradictory and depend on the strain, age, sex, and a number of other experimental conditions [52,53,54,55]. The experiments with the acute immobilization challenge aimed to explore whether chronic isolation in SHRs specifically affects the acute stress response and whether N-Pep-Zn intake can control this response. In 1936, the first scientific article of Hans Selye on ‘general adaption syndrome’ was published in *Nature*, and the data based on experiments in rats that were exposed to severe insults/stressors suggested a ‘nonspecific bodily response’ with three major, grossly visible changes: hyperemia and enlargement of the adrenals, atrophy of the thymus and lymph nodes, and hemorrhagic gastric ulcers (the “stress triad”) [56]. The thymus is the central organ of the immune system; it is essential for the development and maintenance of normal immune system, especially cell-mediated immunity. The neuroendocrine system regulates early T-cell differentiation by the transcription of neuroendocrine genes in the stromal network [57]. Since thymopoiesis is essential for the development and maintenance of a robust and healthy immune system, the thymus size and function change dramatically with age as well as in response to stressors, while acute thymic atrophy is a complication of many infections, environmental stressors, and other clinical conditions [58,59,60]. Complex relations exist among important molecules belonging to the central nervous system and immune system, both in norm and during the chronic stress response [61]. Stress may transform the inflammatory signal of cytokines into a nervous signal (neurotransmitters); in turn, this process uses the endocrine system signals (e.g., cortisol) to counterbalance against the immune system. Such molecular links could explain how stress plays a role in the etiopathogenesis of several diseases through this complex interplay. The microbiota–gut–immune–brain axis was recently postulated to promote mental health or disorders. A dysregulated immune system can shift to an autoimmune response with concomitant neuropsychological consequences in the context of this axis [62].

The isolation stress did not significantly affect the response of glucose and corticosterone to the acute restraint, confirming our above suggestions about effective mechanisms of adaptation in SHRs providing their successful viability in spite of developing gross pathological changes in many systems and organs. The adaptation of the HPA to the conditions of SHR organisms may explain why acute restrained stress did not induce adrenal hypertrophy. However, the response of the thymus turned out to be the weak link, manifesting a pathological influence of chronic isolation stress: unlike the SHRsoc group, the SHRiso group demonstrated acute thymus involution within 1 h of restraint. N-Pep-Zn intake fully prevented the expression of this well-known marker of the response to a severe acute stress (Figure 10). Moreover, the N-Pep-Zn significantly smoothed the response of glucose and cortisol to acute stress in SHRs with chronic isolation experience (Figure 8 and Figure 9). This may indicate an alleviating effect of N-Pep-Zn on both the HPA axis response to acute stress (corticosterone) and sympathetic reactivity (glucose). As was mentioned above, alterations in the amylase activity, especially during stress, also reflect the sympathetic reactivity. Similarly to corticosterone and glucose, neither the SHRsoc nor the SHRiso group responded to the acute restraint with changes in their amylase activity (Figure S1b). However, the amylase activity decreased in the stressed SHRisoP rats as compared to the control SHRisoP animals, which confirms our suggestion about the reduction in sympathetic reactivity to acute stress caused by N-Pep-Zn in animals which have experienced chronic isolation stress. Thus, N-Pep-Zn intake alleviated the response to acute stress by acting on the sympathetic system and HPA axis, both essential first-line systems which provide urgent adaptive signals in response to stress, and protects the thymus from stress-induced involution, thus exerting potential beneficial effects on the immune system.

Since only a few data are available on the effects of N-Pep-Zn (this is the first experimental in vivo study), we can only speculate about the specific mechanisms underlying beneficial stress-protective effects of this peptide. We can only hypothesize which N-Pep-Zn components are most active and how they reach their targets: in general, the intestinal absorption of peptide drugs is severely hindered by both biochemical and physical barriers. Although enzymatic hydrolysis can take place in the gastrointestinal tract, reducing the stability of polypeptides, while the plasma membranes of cells form a selective physical barrier [13], gastrointestinal peptide uptake and the corresponding effects have been widely described [63,64,65,66]. We can currently merely guess about the nature of the specific targets of N-Pep-Zn; however, from the data that have been reported on analogous parenterally administered substances such as cerebrolysin [67] and the parent substance N-PEP-12 [38], we can suggest that these brain-derived peptide preparations exert their neurotrophic factor-like favorable effects on neuroplasticity. Similarly to these peptides, N-Pep-Zn may selectively protect vulnerable limbic structures, specifically the hippocampus, involved in learning, memory, and emotions. In unfavorable conditions, an uncontrolled stress response induces signal transduction, triggering pathways leading to neuroinflammation, neurodegeneration, and, eventually, to cognitive and emotional disturbances. We suggest that N-Pep-Zn is able to modulate the stress response affecting multiple essential systems, promoting adaptation (HPA axis, sympathetic and immune systems) and thus modifying the stress response, shifting it toward more resilient and rational reactions. This potential of N-Pep-Zn, together with a number of other favorable effects on different levels of neuroplasticity described for other brain-derived neuropeptides [38,67], may contribute to enhancing memory and cognitive performance.

## 5. Conclusions

Chronic social isolation accompanied by aging in SHRs induces impairments in cognitive functions (learning and memory) and stress-reactivity. In aged SHRs, N-Pep-Zn significantly improves the effects on spatial learning and memory caused by chronic social isolation. N-Pep-Zn favorably influences the acute response to restraint stress affected by chronic social isolation. Thus, N-Pep-Zn enhances adaptive capabilities towards chronic and acute stress in aged rats with spontaneous hypertension. The improved memory functions in N-Pep-Zn-treated SHRs and the ameliorated response to acute stress suggest that this substance might have benefits in protecting both cognitive function and adequate stress resilience in elderly people experiencing chronic stress (e.g., loneliness and social isolation) as well as the load of usual daily and/or unexpected acute stressors.

## Figures and Tables

**Table 1:** Data of Friedman ANOVA on ranks applied to the latency decrement during the training session in the Barnes maze.

Group	χ^2^	df	*n*	*p*-Value
SHRsoc	16.61	4	16	0.00230
SHRisoC	10.08	4	11	0.03906
SHRisoP	18.42	4	16	0.00102

**Table 2:** Data of Wilcoxon matched-pair test applied to the latencies on day 1 and days 3–5 during the training session in the Barnes maze.

Group	Training Days			
	1–2	1–3	1–4	1–5
SHRsoc	Z(15) = 1.65; *p* = 0.098	Z(15) = 2.25; *p* = 0.024	Z(15) = 3.26; *p* = 0.0011	Z(15) = 3.18; *p* = 0.0015
SHRisoC	Z(10) = 1.02; *p* = 0.31	Z(10) = 2.40; *p* = 0.016	Z(10) = 2.80; *p* = 0.0051	Z(10) = 1.60; *p* = 0.11
SHRisoP	Z(15) = 2.17; *p* = 0.030	Z(15) = 2.95; *p* = 0.0032	Z(15) = 2.84; *p* = 0.0044	Z(15) = 2.90; *p* = 0.0038

**Table 3:** Data of Friedman ANOVA on ranks applied to the latency decrement during the reversal training session in the Barnes maze.

Group	χ^2^	df	*n*	*p*-Value
SHRsoc	3.38	2	16	0.185
SHRisoC	2.84	2	11	0.242
SHRisoP	3.33	2	16	0.189

**Table 4:** Data of Wilcoxon matched-pair test applied to the latencies on day 7 and days 8–9 during the reversal training session in the Barnes maze.

Group	Training Days	
	7–8	7–9
SHRsoc	Z(15) = 2.40; *p* = 0.016	Z(15) = 1.76; *p* = 0.079
SHRisoC	Z(10) = 1.94; *p* = 0.053	Z(10) = 1.96; *p* = 0.050
SHRisoP	Z(15) = 1.59; *p* = 0.11	Z(15) = 1.48; *p* = 0.14

**Table 5:** Data of Friedman ANOVA on ranks applied to the number of working memory errors during the training and reversal training sessions in the Barnes maze.

Group	χ^2^	df	*n*	*p*-Value
Acquisition				
SHRsoc	22.033	4	16	0.0002
SHRisoC	3.74	4	11	0.442
SHRisoP	11.61	4	16	0.020
Reversal				
SHRsoc	0.95	2	16	0.622
SHRisoC	8.061	2	11	0.0178
SHRisoP	2.15	2	16	0.341

## Data Availability

The datasets generated during and/or analyzed during the current study are not publicly available due to the requirements of the Institute of Higher Nervous Activity and Neurophysiology, but are available from the corresponding author on reasonable request.

## Supplementary Materials

The following supporting information can be downloaded at: https://www.mdpi.com/article/10.3390/biomedicines12102261/s1, Figure S1: Effects of acute 1-h restraining on the salivary amylase activity (a) and the blood IL-6 content (b) in the SHRsoc, SHRiso, and SHRisoP groups; Table S1: Main behavioral indices assessed in the Open Field Test, Elevated Plus Maze, Sucrose Preference Test and 3-chamber Social Test; Table S2: Biochemical indices in SHR groups after chronic isolation (36W, Figure 1) and effects of acute restraint stress.

## Abbreviations

CNS	central nervous system
EPM	elevated plus maze
HPA axis	hypothalamo-pituitary-adrenal axis
OFT	open field test
SHR	spontaneously hypertensive rat
SHRiso	SHRs subjected to chronic social isolation
SHRisoP	SHRs subjected to chronic social isolation and receiving N-Pep-Zn
SHRsoc	SHRs housed in groups
SPT	sucrose preference test
SVD	small vessel disease
VCI	vascular cognitive impairment
VCID	vascular cognitive impairment dementia
VD	vascular dementia
