# Supplementary material for: N-Pep-Zn Improves Cognitive Functions and Acute Stress Response Affected by Chronic Social Isolation in Aged Spontaneously Hypertensive Rats (SHRs)
Source: Biomedicines. 2024 Oct 4;12(10):2261. doi: 10.3390/biomedicines12102261 (PMC11503999; doi:10.3390/biomedicines12102261)
Supplement: Supplementary file 1 [file biomedicines-12-02261-s001.zip › biomedicines-3207553-supplementary.pdf]

Supplementary material to the paper "N-Pep-Zn improves cognitive functions and acute stress re-sponse affected by chronic social isolation in aged spontaneously hypertensive (SHR) rats", authors Mikhail Y. Stepanichev, Mikhail V. Onufriev, Yulia V. Moiseeva, Olga A. Nedogreeva, Margarita R. Novikova, Pavel A. Kostryukov, Natalia A. Lazareva, Anna O. Manolova, Diana I. Mamedova, Victoria O. Ovchinnikova, Birgit Kastberger, Stefan Winter and Natalia V. Gulyaeva

Table S1. Main behavioral indices assessed in the Open Field Test, Elevated Plus Maze, Sucrose Preference Test and 3-chamber Social Test

Group<br>Index	SHRsoc<br>n=17	SHRiso<br>n=11	SHRisoP<br>n=15
	Open Field Test		
Distance total, m	19.6 ± 2.3	19.8 ± 1.8	21.9 ± 1.6
Distance in center, m	2.3 ± 0.6	2.1 ± 0.6	2.1 ± 0.3
Distance in periphery, m	11.6 ± 1.3	13.8 ± 1.5	15.3 ± 1.4
Distance center/periphery	0.3 ± 0.1	0.2 ± 0.1	0.1 ± 0.0
Velocity, m/s	0.7 ± 0.1	0.7 ± 0.1	0.8 ± 0.6
Rearing	3.5 ± 1.0	3.0 ± 1.7	2.9 ± 0.6
Latency to start, s	16.1 ± 2.4	19.3 ± 2.8	19.6 ± 4.7
Grooming	1.4 ± 0.7	2.9 ± 1.0	1.3 ± 0.4
Defecation boli	0.2 ± 0.2	1.0 ± 0.5	0.9 ± 0.4
	Elevated Plus Maze		
Open arm duration, s	69.0 ± 10.1	53.4 ± 14.8	60.9 ± 9.1
Close arm duration, s	190.3 ± 9.8	209.9 ± 16.5	202.1 ± 10.1
Open arm entries	2.9 ± 0.3	3.0 ± 0.8	3.4 ± 0.5
Close arm entries	4.6 ± 0.5	4.6 ± 0.7	6.7 ± 0.7
	Sucrose Preference Test		
Liquid, g/100 g BW	11.9 ± 0.6	13.7 ± 0.7	15.7 ± 1.2
Sucrose, g/100 g BW	9.6 ± 0.6	10.9 ± 0.6	12.4 ± 1.0
Sucrose preference, %	93.1 ± 1.3	90.2 ± 3.2	92.7 ± 1.3
	3-chamber Social Test (Trial 1)		
Central alley, s	98.2 ± 19.2	129.2 ± 23.9	182.6 ± 32.8
Stranger 1, s	289.1 ± 40.1	228.1 ± 50.1	234.5 ± 21.5
Empty chamber, s	212.6 ± 47.7	242.7 ± 60.9	182.9 ± 22.7
	3-chamber Social Test (Trial 2)		
Central alley, s	95.1 ± 18.2	99.3 ± 21.6	120.4 ± 33.4
Stranger 1, s	220.4 ± 48.5	333.7 ± 49.2	220.0 ± 41.8
Stranger 2, s	284.6 ± 47.7	167.0 ± 42.5	259.6 ± 39.5

Data are presented as Mean ± s.e.m.

Table S2. Biochemical indices in SHR groups after chronic isolation ("Control", 36W, Fig. 1) and effects of acute restraint stress.

Group	SHRsoc		SHRiso		SHRisoP	
Index	Control n=9	Restraint n=8	Control n=5	Restraint n=6	Control n=7	Restraint n=8
IL-1β, pg/ml *	9.89±3.73	35.29±5.37	12.42±4.91	35.56±9.95	15.29±2.13	35.29±7.96
TNFα, pg/ml*	1.78±1.53	9.31±3.07	2.33±1.51	1.27±1.27	0.29±0.29	5.69±3.46
IL-6, pg/ml*	186.88±54.77	212.14±60.30	16.25±9.44	153.33±33.43	124.38±33.19	81.87±20.70
TGFβ, ng/ml*	6.88±0.71	8.04±1.05	6.74±0.60	6.91±0.86	5.57±0.44	7.76±0.61
CRP, μg/ml*	500.90±46.03	481.0±55.2	428.06±20.36	508.5±22.2	439.44±5.63	469.6±31.20
Amylase, U/μg protein#	6.72±2.62	0.71±0.26	2.06±0.84	4.13±2.07	4.27±1.56	0.76±0.27

Data are presented as Mean ± s.e.m. *, in blood; #, in saliva.

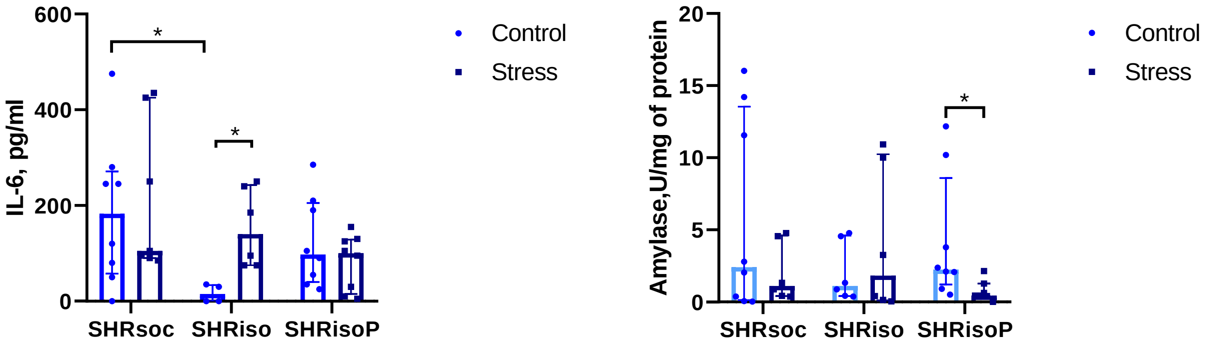

(**a**) (**b**)

Figure S1. Effects of acute 1-h restraining on the blood IL-6 content (a) and the salivary amylase activity (b) in the SHRsoc, SHRiso, and SHRisoP groups. Data are presented as Median ± interquartile range. The differences are significant in at * – $p < 0.05$ according to Mann-Whitney U-test.
